# Structural and functional annotation of the MADS-box transcription factor family in grapevine

**DOI:** 10.1186/s12864-016-2398-7

**Published:** 2016-01-27

**Authors:** Jérôme Grimplet, José Miguel Martínez-Zapater, María José Carmona

**Affiliations:** Instituto de Ciencias de la Vid y del Vino (CSIC, Universidad de La Rioja, Gobierno de La Rioja), Logroño, 26007 Spain; Departamento de Biotecnología, Escuela Técnica Superior Ingenieros Agrónomos, Universidad Politécnica de Madrid, Madrid, 28040 Spain

**Keywords:** Genomic analysis, Gene expression, Grapevine, Phylogenetic analyses, MADS-box genes, *SVP* subfamily, Transcription factors

## Abstract

**Background:**

MADS-box genes encode transcription factors that are involved in developmental control and signal transduction in eukaryotes. In plants, they are associated to numerous development processes most notably those related to reproductive development: flowering induction, specification of inflorescence and flower meristems, establishment of flower organ identity, as well as regulation of fruit, seed and embryo development. Genomic analyses of MADS-box genes in different plant species are providing new relevant information on the function and evolution of this transcriptional factor family. We have performed a true genome-wide analysis of the complete set of MADS-box genes in grapevine (*Vitis vinifera*), analyzed their expression pattern and establish their phylogenetic relationships (including MIKC* and type I MADS-box) with genes from 16 other plant species. This study was integrated to previous works on the family in grapevine.

**Results:**

A total of 90 MADS-box genes were detected in the grapevine reference genome by completing current gene annotations with a genome-wide analysis based on sequence similarity. We performed a thorough in-depth curation of all gene models and combined the results with gene expression information including RNAseq data to clarifying the expression of newly identified genes and improve their functional characterization. Curated data were uploaded to the ORCAE database for grapevine in the frame of the grapevine genome curation effort. This approach resulted in the identification of 30 additional MADS box genes. Among them, ten new MIKC^C^ genes were identified, including a potential new group of short proteins similar to the SVP protein subfamily. The MIKC* subgroup contains six genes in grapevine that can be grouped in the S (4 genes) and P (2 genes) clades, showing less redundancy than that observed in *Arabidopsis thaliana*. Expression pattern of these genes in grapevine is compatible with a role in male gametophyte development. Most of the identified new genes belong to the type I MADS-box genes and were classified as members of the Mα and Mγ subclasses. Ours analyses indicate that only few members of type I genes in grapevine have homology in other species and that species-specific clades appeared both in the Mα and Mγ subclasses. On the other hand, as deduced from the phylogenetic analysis with other plant species, genes that can be crucial for development of central cell, endosperm and embryos seems to be conserved in plants.

**Conclusions:**

The genome analysis of MADS-box genes in grapevine, the characterization of their pattern of expression and the phylogenetic analysis with other plant species allowed the identification of new MADS-box genes not yet described in other plant species as well as basic characterization of their possible role, particularly in the case of type I and MIKC* genes.

**Electronic supplementary material:**

The online version of this article (doi:10.1186/s12864-016-2398-7) contains supplementary material, which is available to authorized users.

## Background

The MADS-box family of transcription factors is present in all eukaryotic genomes analyzed so far, although with higher number of gene members in plant genomes than in other kingdoms. Plant MADS-box genes were initially identified as regulators of flower development but later work showed that they control all major aspects of the life of land plants [1]. This family of transcription factors is defined by the presence of a conserved domain, the MADS-box, in the N-terminal region, involved in DNA binding and dimerization with other MADS-box proteins. Ancestral MADS-box gene duplication predating divergence of plants and animals separated the two main lineages, type I and type II [2, 3], but the presence of around 100 genes in the genomes of angiosperm species suggest that they have considerably expanded in plants [4]. Type II group genes include MEF2-like genes of animals and yeast and MIKC-type genes only found in plants. MIKC-type genes received this name because, apart from the MADS (M) domain, they contain three additional conserved domains, the weakly conserved Intervening (I) domain, the conserved Keratin-like (K) domain and the highly variable C-terminal (C) domain [5] where the latter usually contains conserved subfamily-specific sequence motifs [6]. The I domain is responsible for specificity in the formation of DNA-binding dimers, the K domain mediates dimerization and the C domain functions in transcriptional activation and formation of higher order protein complexes. MIKC-type genes have been further divided in two subgroups, MIKC^C^ and MIKC* based on divergence at the I and K domains and on exon-intron structure [7, 8]. Type I group genes show a simpler gene structure. They are shorter, generally encoding a single exon and lack the K domain.

MIKC^C^-type genes were initially identified as floral organ identity genes in *Antirrhinum majus* and *Arabidopsis thaliana*. Further genetic and molecular analyses grouped their biological functions in flower organogenesis into five classes: A, B, C, D and E, which are required, in different combinations, to specify the identity of sepals (A + E), petals (A + B + E), stamens (B + C + E), carpels (C + E) and ovules (D + E). In Arabidopsis, genes belonging to these functional classes were *APETALA1* (*AP1*) in class A, *PISTILATA (PI)* and *APETALA3* (*AP3*) in class B, *AGAMOUS* (*AG*) in class C [9], *SEEDSTICK/ AGAMOUS-LIKE 1* (*STK/AGL11*) and *SHATTERPROOF (SHP)* in class D [10] and *SEPALLATA* (*SEP1, SEP2, SEP3, SEP4*) genes in class E [11]. MIKC^C^ genes in the *AG* and *APETALA1/FRUITFULL* (*AP1*/*FUL*) subfamilies also participate in fruit and seed development [12–14]. Other MIKC^C^ genes were later identified as involved in different regulatory networks controlling flowering time and flower initiation: *FLOWERING LOCUS C* (*FLC*), *SUPRESSOR OF OVEREXPRESSION OF CONSTANTS 1* (*SOC1*) and *SHORT VEGETATIVE PHASE* (*SVP*) are involved in the regulation of flowering transition by the integration of signals from different flowering time regulatory pathways [15–18]. These genes function as either positive (*SOC1, AGL24*) or negative regulators (*FLC*, *SVP*) of flower meristem identity genes together with other subfamilies such as *AGL15* [19–21], *AGL12* and *AGL17* [19, 22–25].

The MIKC* subgroup (or Mδ group [26]) has a small size in all plant species examined so far, ranging from two genes in the basal eudicot (Eschscholzia) and the basal angiosperm (Aristolochia) [27] to six genes in *Arabidopsis thaliana* [26]*.* MIKC* structure is very similar to MIKC^C^ genes but the K-domain is poorly conserved in its last part and gene structure show an exon duplication in its 5’ region [27]. Phylogenetic analyses of MIKC* genes from a broad variety of vascular plants confirm the existence of two clades (S and P) previously determined in Arabidopsis and rice [28]. In Arabidopsis, MIKC* regulatory function depends on the formation of heterodimers between proteins of the S (AGL66 and AGL104) and P clades (AGL30, AGL65, AGL94). With the exception of AGL67 (S) that seems to be involved in late embryo development [29] these genes are crucial for development of the Arabidopsis male gametophyte [8, 30]. MIKC* genes seem to retain a conserved and essential role in gametophyte development during the evolution of land plants [27].

Type I genes are very variable in number among flowering plants, ranging from 11 to 229 members [4]. In Arabidopsis, this group has 61 members distributed in three subclasses: Mα (25 genes), Mβ (20 genes) and Mγ (16 genes) [26]. Contrary to the evolution of MIKC-type genes, mostly related to genome duplications, type I MADS-box genes seem to be predominantly duplicated via segmental duplications. This idea is supported by their proximity in the Arabidopsis genome and by their phylogenetic analyses in different species, since species-specific clusters of type I genes have been found in many species [4, 31]. Expression of type I Arabidopsis genes in central cell, antipodal cell and chalazal endosperm of the embryo sac, indicate that they play an important role in female gametophyte and early seed development in [32, 33]. Type I proteins [34] interact predominantly among them. Mα-type proteins preferentially form heterodimers with Mβ or Mγ-type proteins whereas interactions within the same subclass are rare.

The genome of the PN40024 grapevine was first established from a 8X assembly [35] and later updated with a 12X assembly available at https://urgi.versailles.inra.fr/Species/Vitis/Data-Sequences from which a gene annotation (VCOST) was performed and is available at the ORCAE platform [36]. The MIKC^C^-type genes were previously analyzed within the 8X version [37] and recently the whole MADS-box gene family was analyzed on an early annotation version (v0) of the 12X assembly [38]. Here, we report a thorough, unbiased identification and analysis of the grapevine MADS-box gene family. This work does not represent a new characterization of an advanced annotation version but we have detected new genes and curated all their sequences. This work also represents a direct application of the published grapevine nomenclature recommendations [39] on the annotation of a complex gene family.

Our analysis has permitted to complete the MIKC^C^-type genes set with the discovery of 10 new genes, mainly belonging to the SVP, AGL17, BSister (BS) and AGL6 subfamilies, as well as the characterization of the complete set of MIKC* and type I MADS-box genes of grapevine.

Phylogenetic analyses of MIKC* and type I MADS-box genes within a wide range of plant species have permitted to infer homologies for key genes that could be involved in gametophytes development. Furthermore, *in silico* analysis of gene expression corroborate the expression patterns described for MIKC^C^-type genes and additionally support the results of the phylogenetic analysis of MIKC* and type I MADS-box genes.

## Results

### MADS-box gene identification, annotation and mapping within the grapevine genome

#### Gene identification and structural annotation

Genes that were previously identified as MADS-box [40] were used to perform sequence comparison analyses, either against the most up to date gene predictions from CRIBI V1 and V 2, the NCBI refseq (on the 12Xv1 of the genome assembly) and the VCOST (on the 12Xv2 of the genome assembly) as well as directly against the reference genome sequence to check whether any potential gene could had been missed by these predictions. In this way, we identified 169 genome regions that shared homology with at least one of the genes. Forty two regions did not contained MADS-box genes since there was no MADS domain. Thirty seven regions had a MADS-box domain but deduced genes did not appear to be functional given their truncation or the presence of stop codons inside the sequence (Additional file 1). In fifteen of them, the non-functional condition might be due to incomplete sequence data in the assembly or to natural genetic variation within grapevine, with the corresponding gene being functional in another cultivar. Finally, a total of 90 MADS-box genes with a functional structure were identified in the grapevine genome (Table 1). Compared with a previous study [38], performed on the Genoscope grapevine annotation v0 version, 37 new genes were detected. Thirty genes were also new when compared to the CRIBI V1 annotation and 19 were new with respect to Refseq (http://www.ncbi.nlm.nih.gov/refseq/). Overall, 14 of these genes had never been detected before in any of the automatic gene model predictions.

**Table 1:** Grapevine MADS-box genes

Locus ID	Position	Locus ID	Position	Locus ID	Position
Vitvi00g00586	chr00:13358491..13359102	**Vitvi00g01439**	chr00:23413171..23413974	**Vitvi00g00267**	chr00:6600239..6600842
Vitvi01g01424	**chr01:18957579..18958217(−)**	Vitvi01g01831	**chr01:19022801..19023439(−)**	Vitvi01g00008	chr01:150565..175325(−)
Vitvi01g00011	chr01:194371..206245(−)	**Vitvi01g01673**	chr01:22436834..22521665(−)	Vitvi01g01677	chr01:22565097..22582170(−)
Vitvi01g00126	**chr01:1353272..1355573**	**Vitvi02g01306**	chr02:13242266..13243303	**Vitvi02g01303**	chr02:13263986..13264774
Vitvi02g00206	**chr02:1905662..1906827**	Vitvi02g00427	**chr02:4010223..4021137**	Vitvi03g00729	**chr03:8696026..8696664**
Vitvi03g00730	chr03:8744237..8744878	Vitvi03g00732	**chr03:8776002..8776646**	Vitvi03g00733	chr03:8793246..8793890
Vitvi03g00734	chr03:8820035..8820694	***Vitvi03g01067***	chr03:16246257..16246517(−)	***Vitvi03g01317***	chr03:16246670..16246942(−)
**Vitvi03g01318**	chr03:16247094..16247366(−)	Vitvi03g01320	chr03:12389395..12426843(−)	Vitvi03g01059	chr03:16006264..16036390(−)
Vitvi03g00819	chr03:10121969..10163135	**Vitvi04g01016**	chr04:14881133..14881462(−)	Vitvi04g00171	chr04:1588465..1591158(−)
***Vitvi04g01404***	chr04:19762228..19764252	**Vitvi05g01725**	chr05:8792525..8793580	**Vitvi05g01726**	chr05:2819956..2820663(−)
**Vitvi05g01728**	chr05:2826143..2826844(−)	**Vitvi05g01729**	chr05:2828896..2829603(−)	**Vitvi05g01727**	chr05:2835002..2835637(−)
**Vitvi05g01730**	chr05:2823195..2823892(−)	***Vitvi07g02071***	chr07:12294351..12294983	Vitvi07g02072	chr07:22189659..22189847
Vitvi07g01441	chr07:19961312..19968599(−)	Vitvi07g01516	chr07:20841683..20858565(−)	Vitvi07g01520	chr07:20874707..20895415(−)
Vitvi07g01792	chr07:23583189..23586056(−)	**Vitvi08g01968**	chr08:19810455..19810682(−)	**Vitvi08g01967**	chr08:4962832..4964430(−)
Vitvi08g01935	**chr08:22162334..22164980(−)**	Vitvi10g00842	chr10:10291733..10292383	**Vitvi10g01589**	chr10:8229666..8230313
**Vitvi10g01588**	chr10:8689978..8690526	**Vitvi10g01593**	chr10:8694120..8694740	**Vitvi10g01592**	chr10:6882283..6882954(−)
**Vitvi10g01591**	chr10:8703104..8703412(−)	**Vitvi10g01590**	chr10:8715924..8716592(−)	Vitvi10g01395	**chr10:19893758..19899254**
Vitvi10g00663	chr10:7289565..7297020	Vitvi12g00019	**chr12:419028..430663**	Vitvi13g01861	chr13:26016002..26024480
Vitvi14g00026	chr14:307222..307932	Vitvi14g01341	chr14:23320536..23341023(−)	Vitvi14g01344	chr14:23363477..23379323(−)
Vitvi14g01526	chr14:25510046..25535960	**Vitvi15g01209**	chr15:10201349..10202245(−)	***Vitvi15g01212***	chr15:13193733..13194680
**Vitvi15g01208**	chr15:14612791..14614029	***Vitvi15g01211***	chr15:13159995..13160894(−)	**Vitvi15g01207**	chr15:14586127..14587383(−)
Vitvi15g01213	chr15:4417318..4417578(−)	***Vitvi15g01210***	chr15:10215314..10222523(−)	Vitvi15g00776	chr15:15406916..15424196(−)
Vitvi15g00774	chr15:15375963..15398782	Vitvi15g01214	chr15:3906158..3907147	Vitvi15g00225	chr15:4852394..4882919
Vitvi16g00898	**chr16:16506874..16543807(−)**	Vitvi16g00894	chr16:16451196..16466066	**Vitvi17g01308**	chr17:18151701..18152690
**Vitvi17g01307**	chr17:9751998..9752990	**Vitvi17g01306**	chr17:8039848..8040840(−)	Vitvi17g00470	chr17:5565954..5584339(−)
Vitvi17g00471	chr17:5589790..5596034(−)	Vitvi17g00098	**chr17:1009821..1012257**	Vitvi17g00614	chr17:7012571..7017470
Vitvi18g00221	**chr18:2291990..2293992(−)**	Vitvi18g02133	**chr18:30305897..30312249(−)**	Vitvi18g01044	chr18:11506619..11512146
Vitvi18g02145	chr18:30685886..30701598	Vitvi18g00361	**chr18:4014840..4017395**	Vitvi18g00517	chr18:5694915..5711044
Vitvi18g00553	chr18:6351256..6376057	Vitvi18g00700	chr18:7968590..7971054	**Vitvi19g01487**	chr19:19514486..19515085
**Vitvi19g01785**	chr19:19540899..19541471	**Vitvi19g01491**	chr19:19578381..19581226(−)	Vitvi19g00027	chr19:329777..330095

Bold Locus ID: New genes compared to Wang et al. [38] found in the v0 annotation. Italic bold locus ID: New genes compared to v1 annotation. Bold position: The structure of the CDS was curated compared to v0

Gene models were curated using the data collected from gene structure comparisons as well as the available RNAseq data from our laboratory ([41], V. Grbic, P. Carbonell personal communication) to validate actually expressed exons. These data, that included unpublished work, were particularly valuable in the present context since they include expression information of reproductive organs that are susceptible to show expression of MADS-box genes. This data also allowed evaluating the expression of newly detected genes, not represented in microarray data, by redoing the bioinformatics analysis of original RNAseq data with an updated GFF file. Gene structure described [38] was confirmed for 38 genes. One of them previously allocated to the unknown chromosome was discarded because it already existed on chromosome 15. The structures of 15 other genes were curated. Data relative to the detection of the MADS-box genes in older genome annotations or gene-sets are summarized in Additional file 2.

### Gene nomenclature

To clarify gene nomenclature, we built a phylogenetic tree of the MADS-box protein coding genes in grapevine and Arabidopsis (Additional file 3) as recommended by the Super-Nomenclature Committee for Grape Gene Annotation (sNCGGa) [39]. Within type I and type II MADS-box genes, 42 grapevine genes correspond to MIKC^C^ genes (previously we had identified 32 of them [37]), 6 to MIKC* genes (or Mδ-type), 23 to Mα-type I and 19 to Mγ-type I genes. No Mβ-type I genes seem to be present in grapevine (Additional file 3). For MIKC^C^ genes, when the gene had already been described and the symbol fit the recommendations of the sNCGGa, the same symbol was conserved. For genes that had not been described before and had an Arabidopsis ortholog, Arabidopsis gene symbol was used. For the rest of the genes, the symbol was composed of the subfamily symbol and a number or a letter to differentiate the different members. For the MIKC^C^ genes previously identified [37], symbols were kept with minor corrections (e. g. *VviSOC1.1* is now called *VviSOC1a*). Symbols used in [38] were not kept because they were attributed according to their respective chromosome position, which made no more sense with the inclusion of 37 additional genes and because that system was not functional (e.g. the symbol *VviAGL15a* indicates that the gene is a member of the subfamily AGL15 of the MIKC^C^ while M*ADS25* gives no information). The new *VviSVP*-like sequences were designed *VviSVPS1* to 5. Following the recommendations of the sNCGGa, we named the MIKC*-subgroup (or Mδ) as MADSD; the Mα-type I subclass as MADS1A and the Mγ-type I subclass as MADS1G. Within the MADSD we distinguished three clades, two corresponding to the previously described S and P clades [27] and a third one. Consequently gene clades were designed as MADSD1, 2 and 3 and the individual genes were discriminated with a letter attributed randomly. Within the MADS1A we distinguished three clades that were designated as MADS1A1, MADS1A2 and MADS1A3. Within the MADS1G we distinguished also three clades denominated MADS1G1, MADS1G2 and MADS1G3. Additional nomenclature details will be presented within the phylogenetic analysis.

### Chromosomal location of grapevine MADS-box genes

**Fig. 1:**
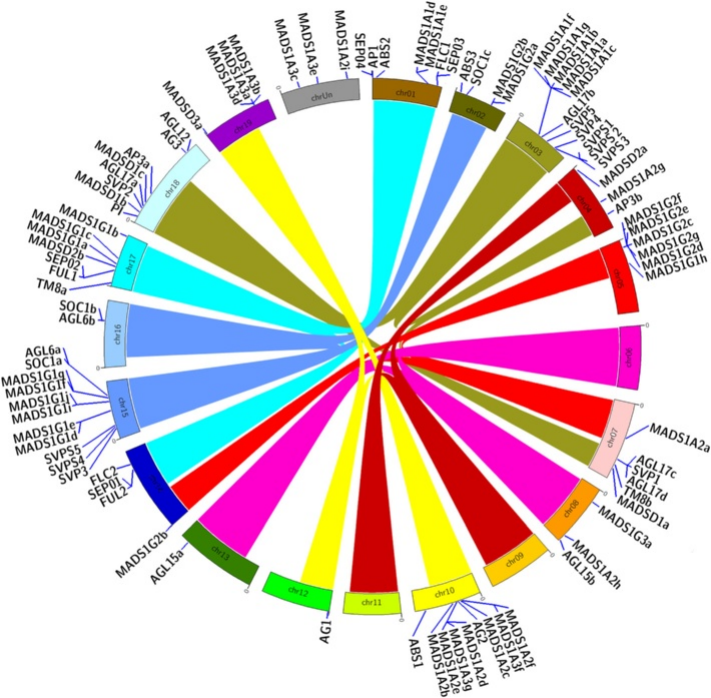
Chromosomal location of grapevine MADS-box genes. Links with the same colors between chromosomes show paralogous regions as previously defined [35]

MADS-box genes were located on 17 of the 19 grapevine chromosomes (Fig. 1, exact position in Table 1), although there are still three genes located in the unknown chromosome. The ten new MIKC^C^ genes compared to [37] were definitely positioned: *VviBS3* on chromosome (chr) 2, *VviSVP3*, *VviSVPS4* and *VviSVPS5* on chr 15, *VviAGL6b* on chr16, *VviAGL17c*, *VviAGL17d* and *VviTM8b* on chr 7, *VviAGL17b*, *VviSVP4*, *VviSVP5* and *VviSVPS1*, *VviSVPS2* and *VviSVPS3* on chr3. In addition, 4 genes located in unknown regions in the previous report were also mapped. As shown in Fig. 1, different members of several subfamilies are located in chromosomal regions that might represent paralogous segments resulting from ancestral polyploidization events [35, 42]. Notably, there are genes from subfamilies SVP and AGL17 located in all 3 paralogous regions on chr03, chr07 and chr18. Genes *VviAG1* and *VviAG2* were located on paralogous regions on chr10 and 12. Subfamilies FLC and SEP had gene members on paralogous regions of chr14 and chr01. The MIKC* subgroup (MADSD genes) were also found in two paralogous segments on chr18 and 07. Within the type I, a cluster of the Mγ clade 2 genes (*MADS1G2c, MADS1G2d, MADS1G2e, MADS1G2f* and *MADS1G2g*) on a region of chr05 was paralog to a region of chr14 that contains the *MADS1G2b* gene. The Mγ-type I subclass also showed two gene clusters on paralogous regions of chr 2 (*MADS1G2a* and *MADS1G2b*) and chr15 (*MADS1G1f*, *MADS1G1g*, *MADS1G1e*, *MADS1G1d*, *MADS1G1i* and *MADS1G1j*).

### Gene structure and phylogenetic analysis of MADS-box genes in grapevine

**Fig. 2:**
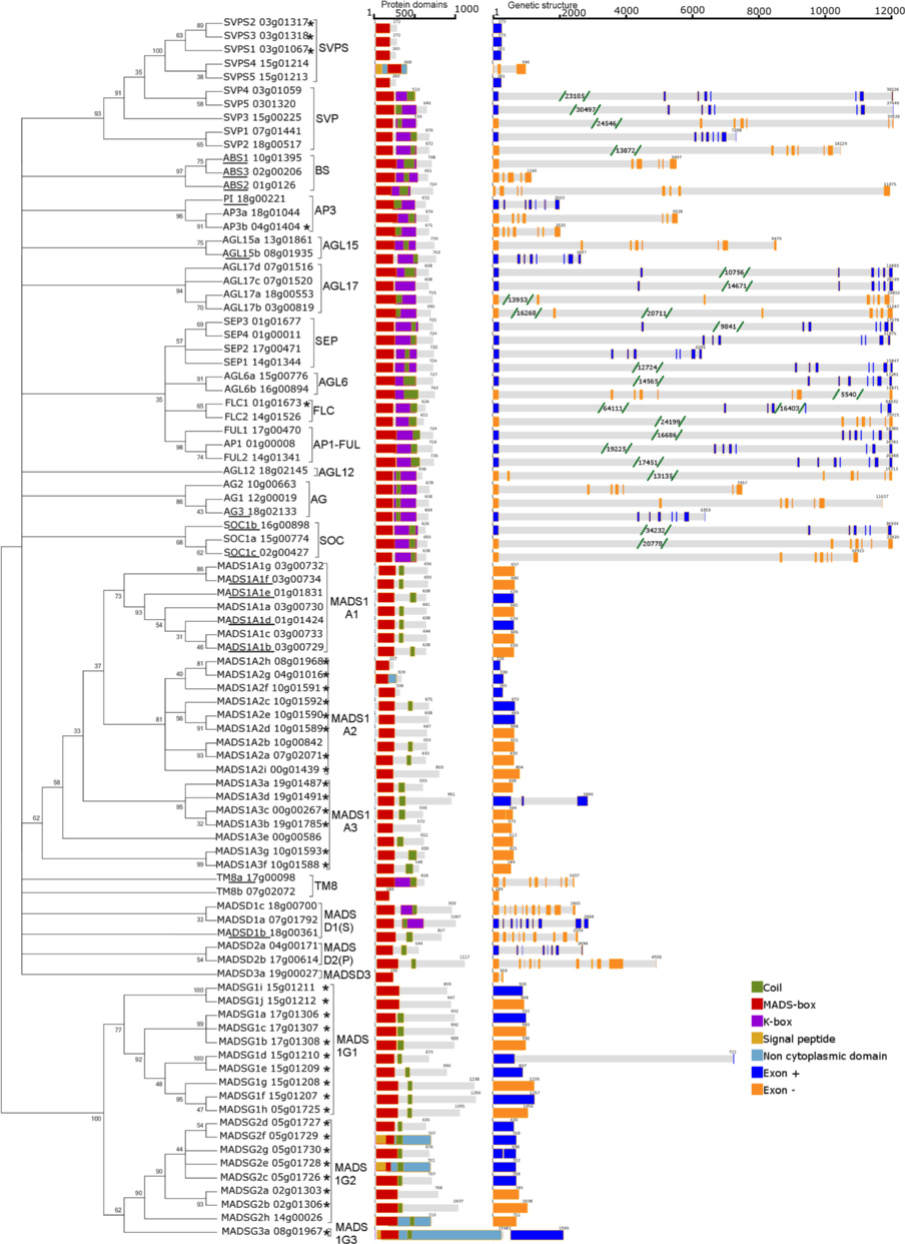
Phylogenetic tree and structure of MADS-box proteins in grapevine. The percentage of trees in which the associated genes clustered together is shown next to the branches. Exons in orange are in the + strand. Exons in blue are in the - strand. * New gene compared to Wang et al. [38]. *Underscore*: gene sequence was curated

**Fig. 3:**
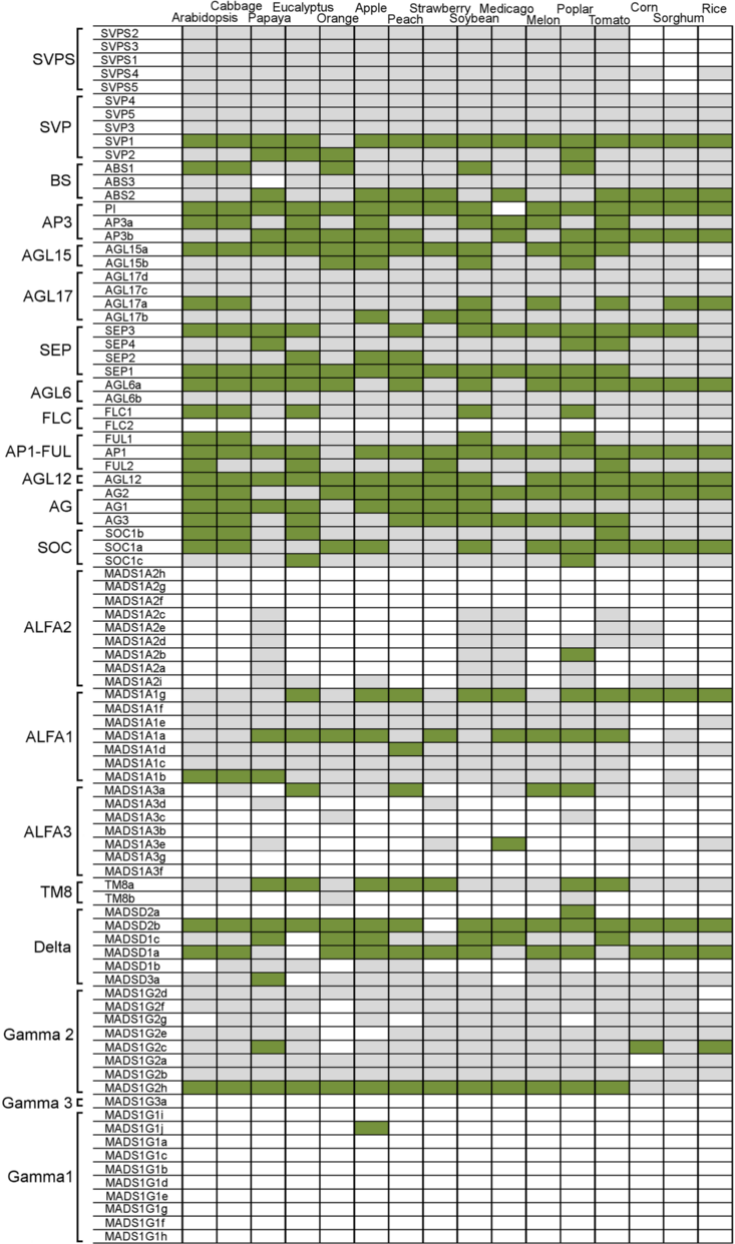
Grapevine MADS-box orthology against plant species with sequenced genome. *Green*: a one-to-one ortholog in the species (ortholog one-to-one = best match in the species that has the grapevine deduced protein as the best match in grapevine.). *Grey*: the grapevine deduced protein has homology in the species genome but no ortholog one-to-one (the best match do not have the grapevine deduced protein as best match). *White*: no match in the species

A phylogenetic analysis was conducted to determine the composition of gene subfamilies, subclassses and clades and to support the establishment of gene nomenclature. Coupled with the identification of protein domains and gene exon structure, we validated in grapevine observations described in other species, in terms of exon/intron structures or locus length. Our structure curation did not result in contradiction to the conclusions of previous works [37, 38] and gene nomenclature was significantly improved (Fig. 2). We also performed an orthology analysis by sequence comparison between grapevine MADS-box deduced proteins and proteins from 16 plant species retrieved at http://planttfdb.cbi.pku.edu.cn in order to obtain information about the possible function of those genes (Fig. 3). The objective was to identify the sequences that show orthology in a given species with a minimal level of complexity (one ortholog in each species, or orthology one-to-one as previously described [43]).

### Update on MIKC^C^ MADS-box genes

**Fig. 4:**
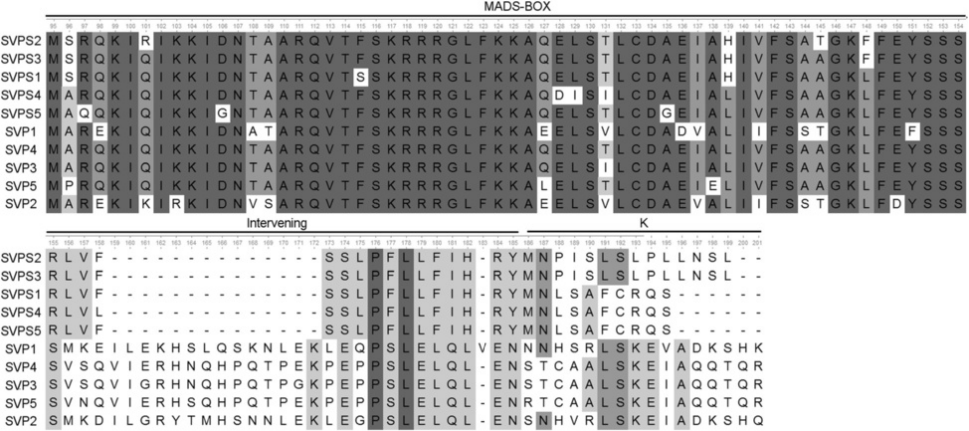
Alignment of the grapevine VviSVP and VviSVPS protein sequences. VviSVP sequences are shown truncated to the area sharing similarity to the VviSVPS

Compared to our previous study we were able to identify 10 new MIKC^C^ genes. Among them, one belonged to the BS subfamily, 2 to the AGL17 subfamily and 1 to the AGL6 subfamily. As described before for MIKC^C^-type II genes they all included the I, K and C domains in addition to the MADS-box. The number of exons was relatively large (average of seven exons per locus) and the loci were much longer than type I genes. In addition to those four new genes, we detected one gene,*VviTM8b*, which appeared related to *VviTM8a* and 5 new gene sequences closely related with the VviSVP subfamily. These genes were called as *VviSVPS* for *SVP Short* and were located on chr03 (3) and chr15 (2) (Fig. 2). The *VviTM8b* gene did not fit the MIKC^C^ type II model, it only contained the MADS-box and it is unclear whether it is expressed and functional. Regarding *VviSVPS*, all but *VviSVPS4*, had only one exon encoding a MADS-box followed by a short amino acid sequence of about 30 amino acids that was different between *VviSVPS2* and *VviSVPS3* on one side and *VviSVPS1*, *VviSVPS4* and *VviSVPS5* on the other side (Fig. 4).

The results of the orthology analyses indicated that *SVPS* sequences were not present in the monocot species considered. In addition, for some genes such as *VviSVP1*, *VviPI*, *VviAGL15a* (except for monocot) and *VviSEP1* (not in monocot) there was only one detected gene in all the species. Interestingly, the new grapevine genes found in the subfamilies BS (*VviBS3*), AGL17 (*VviAGL17c, VviAGL17d*), AGL6 (*VviAGL6b*) and TM8 (*VviTM8b*), did not showed orthology with other species.

### Phylogenetic analysis of MIKC* and type I MADS-box genes in plants

**Fig. 5:**
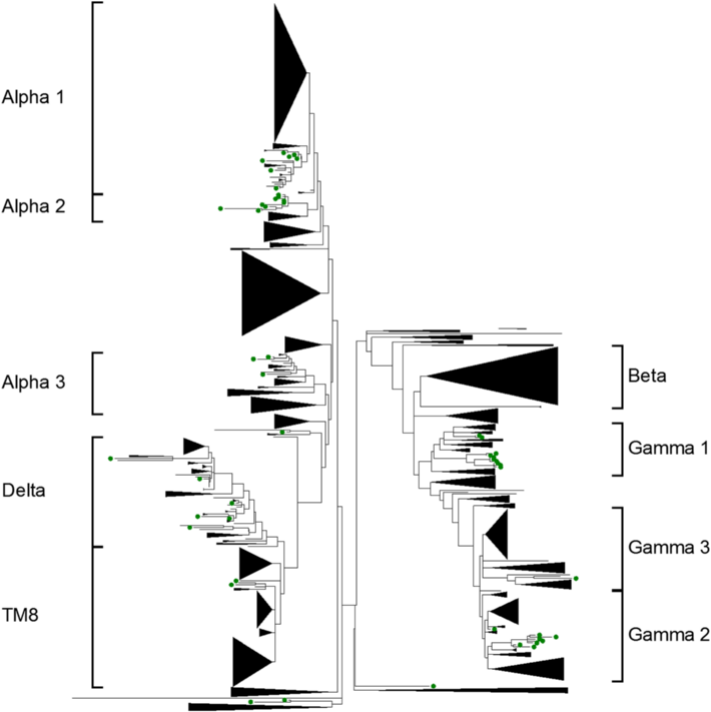
Phylogenetic tree of MIKC* and type I MADS-box proteins in plants. *Green*: grapevine deduced proteins. Branches without grapevine proteins were collapsed

The phylogenetic analysis for MIKC* and type I MADS-box genes from grapevine and Arabidopsis showed many clades of genes that appeared specific to grapevine, thus revealing little information on their possible roles. For this reason, a phylogenetic analysis including MIKC* and type I genes from 16 other plant species was carried out in order to provide additional information on their evolutionary history and possible function and to identify clades and subclades (Fig. 5). Additionally, we also performed an orthology analysis for MIKC* and type I MADS-box genes as described for the MIKC^C^ genes (Fig. 3).

### The MIKC* MADS-box genes

**Fig. 6:**
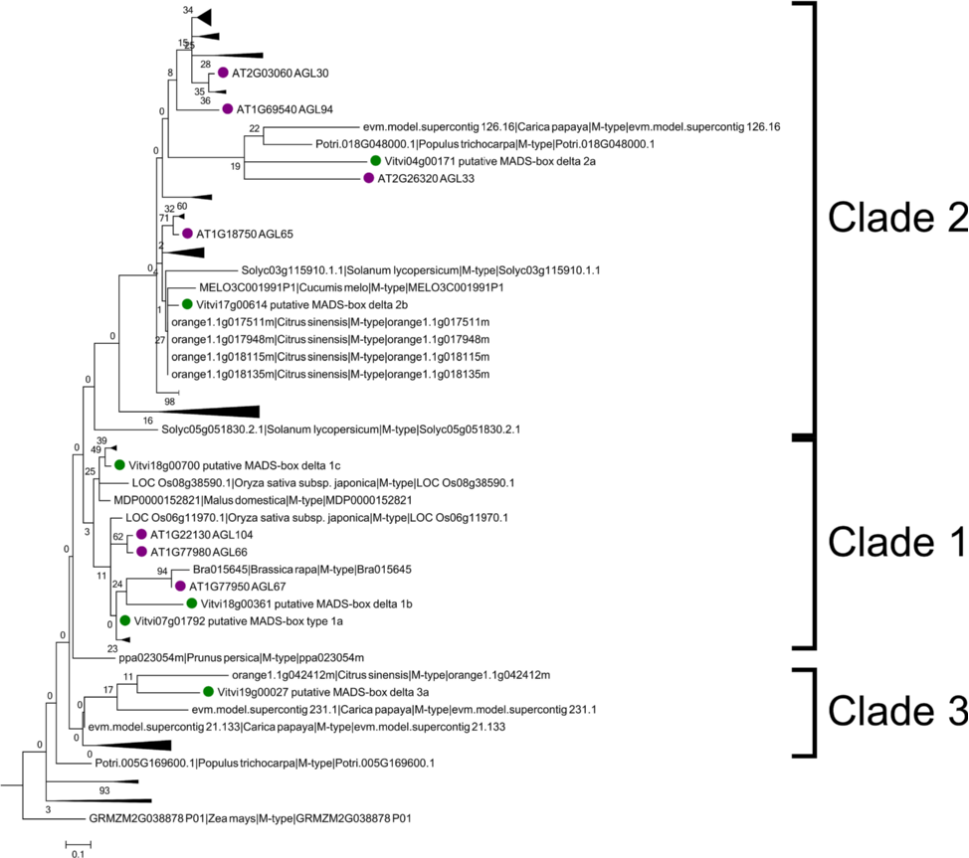
Phylogenetic tree of the MIKC*) MADS-box proteins in plants. *Green*: grapevine deduced proteins. *Pink*: Arabidopsis deduced proteins

Six members, as in Arabidopsis, were detected in the grapevine MIKC*subgroup (Fig. 6). Their gene structure shared with Arabidopsis genes the presence of a higher number of exons (around ten) compared with MIKC ^C^ genes (around 8), although the total lengths of loci were shorter. Two (*MADSD1a*, *MADSD1c*) have an identifiable K domain, but this box could not be detected in the rest either with prosite or pfam (Fig. 2). The two clades previously described in vascular plants [27] were also maintained in grapevine. However, we identified a short protein (*MADSD3a*) not belonging to any of them, that only contained the MADS-box region followed by five amino acids. Clade 1 (S) contained three homologs to *AGL66, AGL67* and *AGL104* (*MADSD1a, MADSD1b* and *MADSD1c*) with *MADSD1a* and *MADSD1b* being the nearest to AGL67. Clade 2 (P), contained two homologs to Arabidopsis *AGL30*, *AGL94* and *AGL65*. This clade 2 also included *AGL33* that seems to show low homology with several proteins in other species including grapevine (*MADSD2a*). *AGL33* is a short sequence without K-domain and there is no evidence of orthology with *MADSD2a*. Grapevine MIKC* genes displayed orthology one-to-one with MADSs in other species (Fig. 3), the most consistent were *MADSD2b* which showed one single orthologs in every species except strawberry as well as *MADSD1a* and *MADSD1c. MADSD2a*, only showed one-to-one orthology with a poplar protein and *MADSD3a* with papaya protein but had homologs in most analyzed species.

### Type I MADS-box genes

Type I genes were characterized by the presence of a single exon. Only *MADS1A3d* had three exons and *MADS1G1d* had two, however we cannot exclude that this could be due to incorrect genome assembly. Within type I genes, it was hard to find clear orthologies between Arabidopsis and grapevine genes, most notably in the Mγ subclass (Fig. 3). Regarding Mβ genes, our analysis revealed that this subclass seems to be absent in grapevine.

### Analysis of the Mα-type I genes

Three clades could be distinguished in the Mα subclass based on phylogeny, Mα1, Mα2 and Mα3. These clades showed similarity to the previously defined groups [32].

**Fig. 7:**
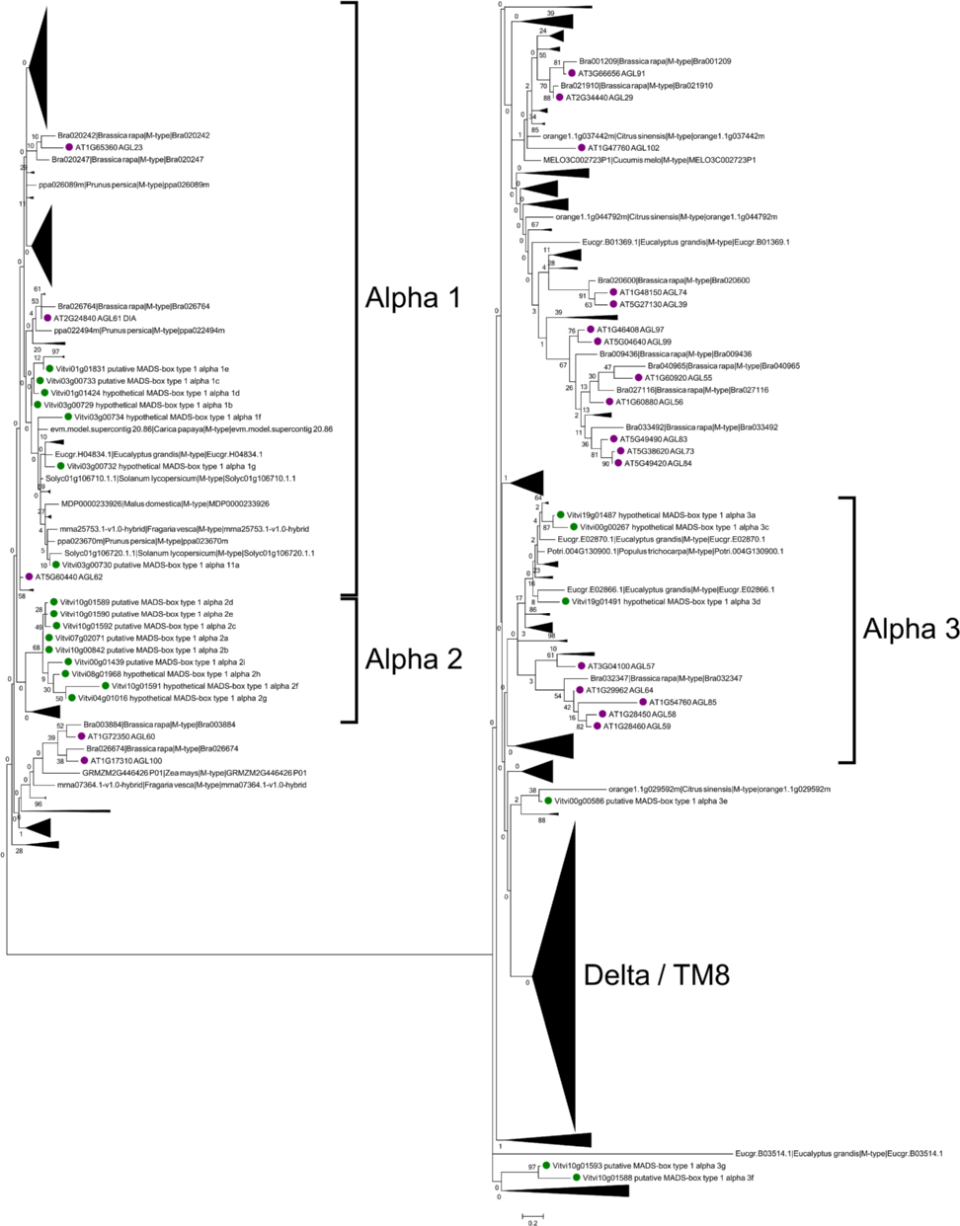
Phylogenetic tree of the Mα-type I proteins in plants. *Green*: grapevine deduced proteins. *Pink*: Arabidopsis deduced proteins

The Mα1clade contained 7 grapevine genes (*MADS1A1a*, *MADS1A1b*, *MADS1A1c*, *MADS1A1f* and *MADS1A1g* on chromosome 3 and *MADS1A1d* and *MADS1A1e* on chromosome 1) (Fig. 7). The clade also included genes from papaya, apple, peach, tomato and Arabidopsis (genes *AGL62, AGL40, AGL23* and *AGL61*). Homology with other species was maintained in this clade, although it was weaker with monocots (Fig. 3). Only *MADS1A1d* and *MADS1A1g* showed monocot homologs. *MADS1A1g* was also the member with most orthologs including monocots, stressing its possible conserved role. Several genes from other species shared higher homology with grapevine members of this clade than the homology detected amongst grapevine genes. These results suggest that duplication of some members of this family is relatively old predating species divergence.

The Mα2 clade grouped all nine grapevine genes in a single subclade with no gene from the other analyzed species (Fig. 7). Genes from other species were detected in this clade but no other species showed this level of duplication, Medicago had three genes and strawberry and soybean had two (Additional file 4). Consistently with those results, the homology detected with genes from other species was weak (Fig. 3). *MADSA2f*, *MADSA2g* and *MADSA2h* were absent in other species and the rest of MADSA2 only shared homology with soybean, Medicago, poplar and papaya. A possible ortholog of *MADSA2b* could only be detected in poplar, which might be a false positive.

Within Mα3 clade, grapevine *MADS1A3* genes generally were distant from each other. A group of three genes (*MADS1A3a, MADS1A3c* and *MADS1A3d*) seemed to share similarity with seven eucalyptus genes and a few others from *Rosacea* (Fig. 7). The other Mα3 genes were much more distant in the phylogenetic analysis and appeared unrelated. Two other genes (*MADS1A3g* and M*ADS1A3f*), seem to be related with a group of nine tomato genes (Additional file 4). *MADS1A3e* was poorly related to any other Mα except a gene from orange trees. Finally, *MADS1A3b* did not group within the Mα subclass in the species analysis (Fig. 6). In summary, within the Mα3, not much homology was detected between grapevine genes and other species genes appearing in this clade including Arabidopsis *AGL57, AGL64, AGL85, AGL58* and *AGL59. MADS1A3a* and *MADS1A3e* were the only members for which orthologs one-to-one could be detected in species other than Arabidopsis (Fig. 3).

### Analysis of the Mγ-type I genes

**Fig. 8:**
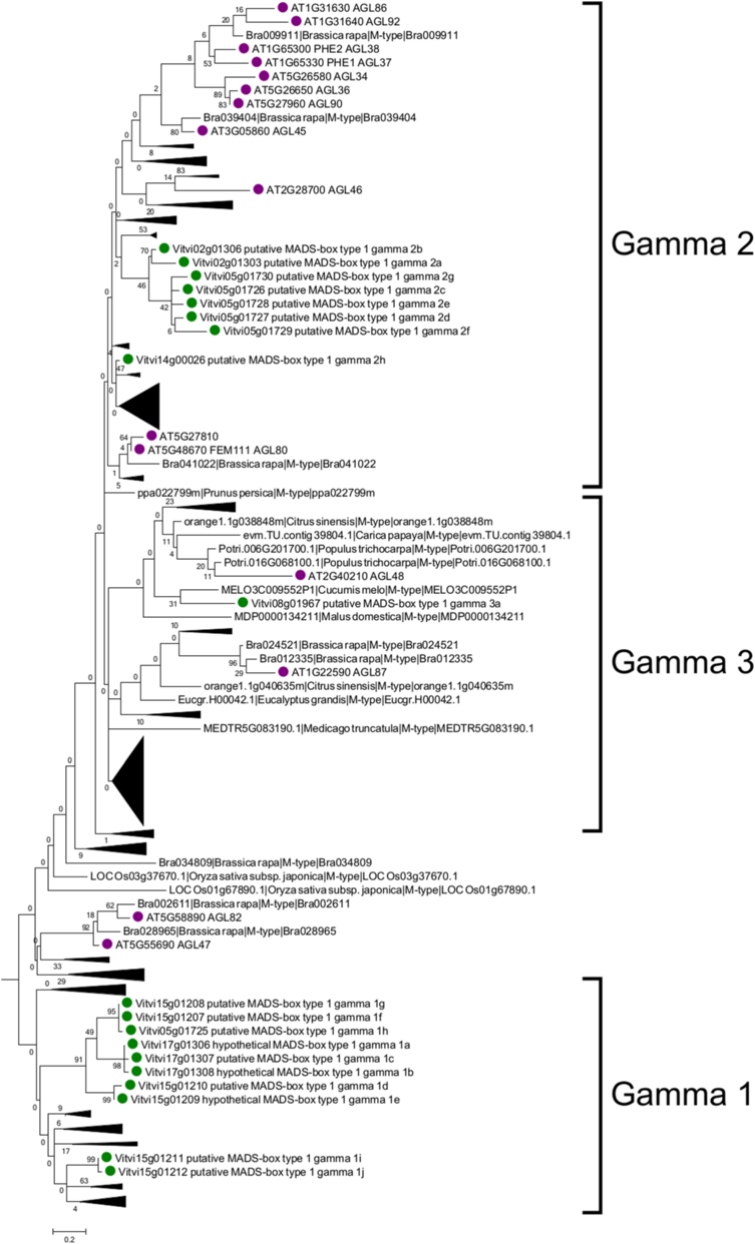
Phylogenetic tree of the Mγ-type I proteins in plants. *Green*: grapevine deduced proteins. *Pink*: Arabidopsis deduced proteins

Three clades could be distinguished within the Mγ subclass, Mγ1, Mγ2 and Mγ3. The Mγ1 clade contained two groups of grapevine genes (Fig. 8). The first group of eight genes was located on chromosome 17 (*MADS1G1b, MADS1G1a* and *MADS1G1c*), chromosome 15 (*MADS1G1d, MADS1G1e, MADS1G1g* and *MADS1G1f*) and on chromosome 5 (*MADS1G1h*). These genes formed a grapevine-specific group with no clear homology to genes from other species. The second group contained two grapevine genes located on chromosome 15 (*MADS1G1i* and *MADS1G1j*) as well as genes from Medicago, soybean and some of the Rosaceae species. *MADS1G1j* showed orthology with apple, this was the only homology with other species detected in the Mγ1 clade (Fig. 3).

Within the Mγ2 clade there was a group of seven grapevine genes, five in chromosome 5 (*MADS1G2c, MADS1G2d, MADS1G2e, MADS1G2f* and *MADS1G2g*) and two on chromosome 2 (*MADS1G2a* and *MADS1G2b*) (Fig. 8). These genes had homologs in all the species analyzed but only *MADS1G2c* showed orthology with papaya and monocot-specific genes (Fig. 3). The closest genes in the phylogenetic tree correspond to a group of apple genes (Fig. 8), which suggests that in both of these fruit species the duplication occurred recently. The Mγ2 clade also included the *MADS1G2h* grapevine gene located on chromosome 14, which seemed to belong to a group of genes that duplicated and expanded in legumes (soybean and Medicago). *MADS1G2h* was identified as one-to-one ortholog of *AGL80*, one of the best characterized Arabidopsis type I genes in this subclass, although *AGL80* appeared in a separated branch in the Mγ2 clade. In this context, both eucalyptus and cabbage contained homologous genes to *AGL80*, probably due to conservation of this gene function along evolution.

Finally, the Mγ3 clade only contained a single grapevine gene (*MADS1G3h*) that was associated with a single gene from melon (Fig. 8). No orthologs could be detected for this Mγ3 grapevine gene.

Globally, the large clades for Mα3 and Mγ1-type I grapevine genes seemed to be mostly specific for grapevine. In general, there was very little orthology one-to-one detected for the MIKC* and type I MADS-box genes with the notable exceptions of *MADSD2b* (only absent in strawberry) and *MADS1G2h* (absent in the tested monocots). In addition, *MADS1A1a, MADS1A1g*, *MADSD1a* and *MADSD1c* were present in about 50 % of the species analyzed.

### Expression analysis of MADS-box genes

**Fig. 9:**
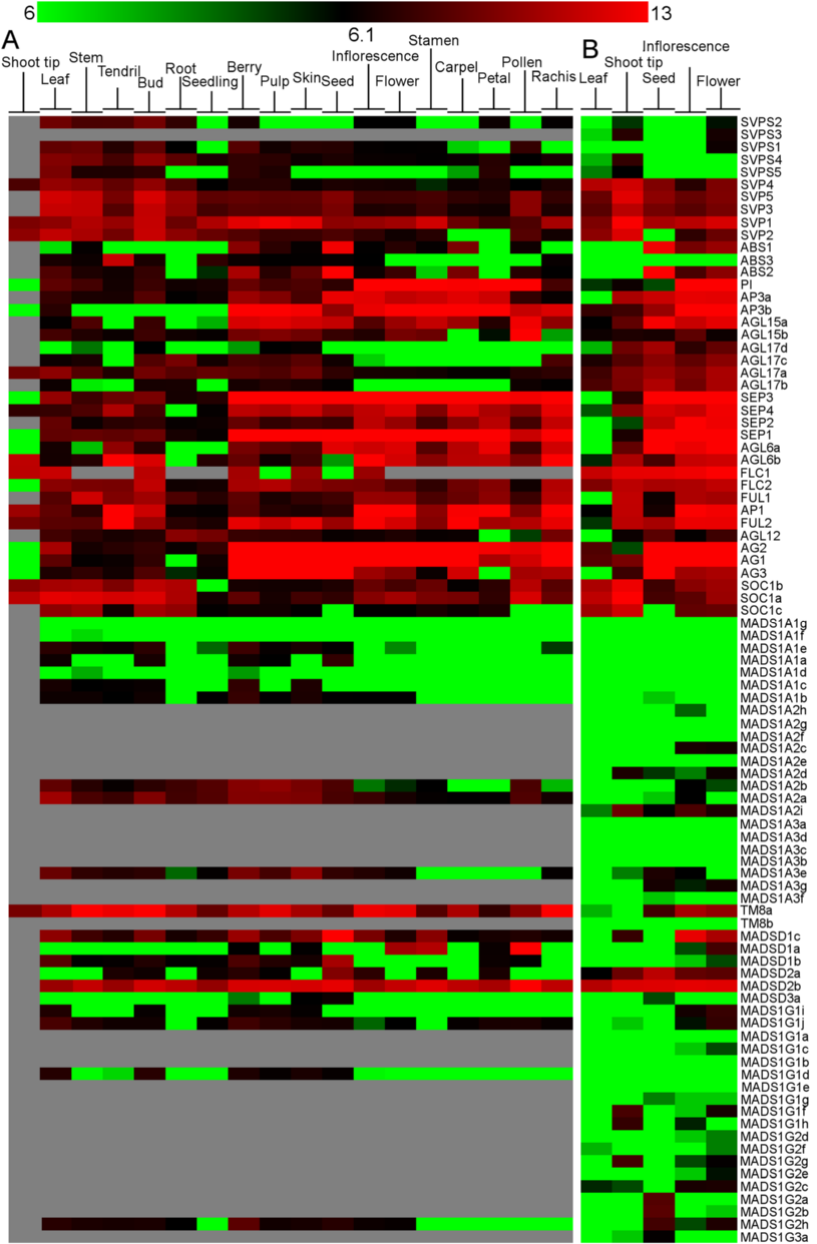
Expression data of the grapevine MADS-box genes per tissues. Color represents the absolute intensity value of expression (log2) in each condition. *Green*: the expression cannot be distinguished from the background noise; *Black*: expression was different from the background noise but not enough to attribute a Plant Ontology; *Red*: gene is expressed. *Grey*: not measured. **a** Nimblegen microarray data **b** RNAseq data

**Fig. 10:**
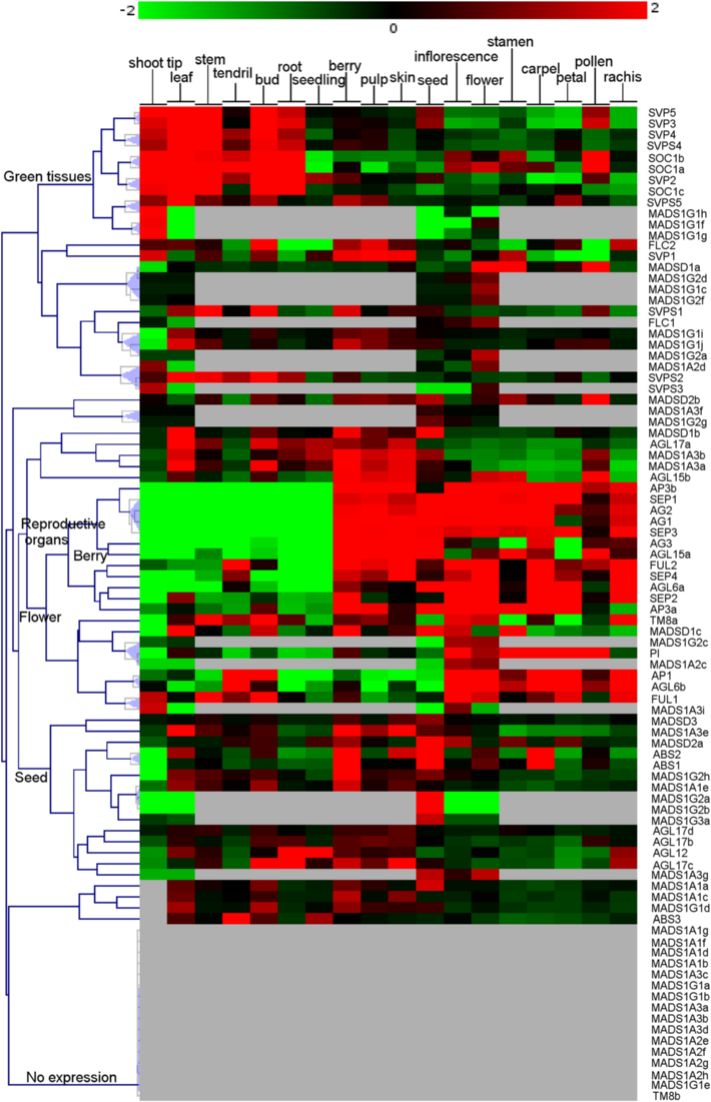
Coexpression tree of the MADS- box genes. *Blue*: GeneChip, *Yellow*: GrapeGen, *Purple*: Nimblegen, *Brown*: RNAseq. Complete data with condition per platform. Color represents the relative difference of expression (log2) between the condition and the average of the values within the same platform

Two types of expression analyses were performed. In the first analysis the objective was to construct an atlas of expression of the MADS-box genes based on the absolute value of gene expression in public data. The results of this study are presented in Fig. 9 that displays the data extracted from the published grapevine gene expression atlas [44] and data available in our laboratory from different RNAseq experiments. In addition, we intended to detect expression in the experiments listed in Additional file 5. When a gene was clearly expressed in a given tissue a Plant Ontology (PO) was attributed to the gene and reported in Additional file 2 and in the ORCAE database. Secondly, we performed a co-expression analysis based on the same original data but using the relative values of expression, centered on the average expression in each platform. The objective here was to determine expression patterns and to identify genes within subfamilies that were following the same pattern and that could be under the same regulatory elements. The results are presented in Fig. 10. The original data used are reported in Additional file 6.

### Expression of MIKC^C^ genes

Expression studies of MIKC^C^ confirmed previous results for those genes previously known [37, 38]. Regarding the new genes found in this study, *VviBS3* showed a pattern of expression in tendrils and buds, quite different to the expression pattern of *VviBS1* and *VviBS2* (berry, seed and flower carpel); *VviAGL17d* showed a pattern similar to *VviAGL17b*, restricted to seeds and flowers whereas *VviAGL17c* expression was detected in roots, berries, seeds, flowers and rachis; *VviAGL6b* expression was detected in shoot tip, tendril, bud, berry, inflorescence, flowers and reproductive organs in general while *VviAGL6a* expression was more restricted to the reproductive phase.

*VviSVPS* sequences showed specific pattern of expression that is different from the *VviSVP* genes in grapevine. *VviSVPS4* expression was similar to *VviSVP4* and *VviSVP5* but it was neither expressed in reproductive organs nor in seeds. *VviSVPS5* showed expression in vegetative organs but also in berry and petals. *VviSVPS1* expression was detected in leaf, buds but also in berry, flower and pollen. *VviSVPS2* showed expression in the vegetative tissues and flowers and *VviSVPS3* in shoot tip and flowers. No expression was detected for *VviTM8b*.

### Expression of MIKC* genes

Expression patterns for genes in clades 1 and 2 were partially overlapping. Within clade 1, *MADSD1a* was expressed in flower and pollen, *MADSD1b* was expressed in leaf, bud, berry, seed and flower and *MADSD1c* was expressed in leaf, bud, berry, seed, inflorescence and flower. Within clade 2, *MADSD2a* showed expression in leaf, root, berry and seed while *MADSD2b* was expressed in bud, berry, seed, stamen and pollen. Finally, *MADSD3a* expression was detected in berry and seed.

### Expression of type I genes

Most Arabidopsis type I genes showed very little expression that is restricted to very specific cells and tissues and limited time span, such as the female gametophyte or specific cells within the developing seed. Transcriptome-wide studies conducted in grapevine have yet to address these tissues since for many of these Type I MADS-box genes we could not detect proof of expression.

### Expression of Mα-type I genes

Most of the Mα1 clade genes did not show expression over the background noise in the microarrays data to clearly state that they are expressed in any tissue. However, *MADS1A1a*, *MADS1A1c* and *MADS1A1e* seemed to have a slight expression in a few tissues and were considered as expressed. This means that they were qualified as “putative genes” in their description in the ORCAE database and in Additional file 2, as opposed to “hypothetical” for genes with no proof of expression [39]. The gene that is conserved among different species, *MADS1A1g*, seemed not expressed either or expression could not be detected. Regarding the Mα2 clade, three genes showed expression in specifics tissues. *MADS1A2a* was allocated to 11 Plant Ontology terms related to vegetative as well as reproductive tissues (Additional file 2). *MADS1A2b* was allocated to 7 Plant Ontology terms related to vegetative as well as reproductive tissues. *MADS1A2i* expression was detected in shoot tips and inflorescences. Both *MADS1A2b* and *MADS1A2c* seemed to have a light expression and were classified as “putative”. Finally, in the Mα3 clade, only *MADS1A3e* was clearly expressed in leaves and berries whereas *MADS1A3f* and *MADS1A3g* were slightly expressed and were classified as “putative” genes. Neither *MADS1A3a*, *MADS1A3b* and *MADS1A3d*, all on chromosome 19, nor *MADS1A3c* were expressed. The closest homologous genes in Arabidopsis (*AGL57*, *AGL58*, *AGL59* and *AGL64*) were expressed in the embryo and peripheral endosperm [32].

### Expression of Mγ-type I genes

In this subfamily, expression was not detected only for three genes in the first clade (Mγ1), *MADS1G1a*, *MADS1G1b* and *MADS1G1e*. For the rest of the genes, *MADS1G1c* and *d* seemed weakly expressed. *MADS1G1f, MADS1G1g, MADS1G1h* and *MADS1G1j* were expressed in leaves (*MADS1G1*j in senescing leaves). In addition, *MADS1G*1j and *MADS1G1i* were expressed in flowers.

The rest of genes in the Mγ2 clade showed very low expression and this was only clearly visible for *MADS1G2a*, *MADS1G2b* and *MADS1G2h* in seeds. Gene *MADS1G3a*, in clade Mγ 3, did not show clear expression in any tissue.

### Coexpression of MADS-box genes and relative expression

Several groups of genes present a high level of expression correlation between each other (distance threshold <0.15). Based on “guilt-by-association” concept, genes with similar expression are likely involved in the same process.

We identified four groups of consecutive co-expressing genes that might be under the expression of the same regulators such as *MADS1G1f* and *MADS1G1g* or *MADS1G1i* and *MADS1G1j* that also coexpressed with *MADS1G2a*. These genes were in a paralogous chromosome segment [35]. This was the only occurrence where genes from the same duplicated region (potential paralogs) were co-expressed. *VviSVPS2* and *VviSVPS3* co-expressed also with *MADS1A2d. MADS1G2a* and *MADS1G2b*, they also co-expressed with *MADS1A1a*.

Interestingly, in the case of *VviSVP5* co-expressing with *VviSVP3* and *VviSVP4* co-expressing with *VviSVPS4*, they are consecutively located in the following order *VviSVP3*, *VviSVPS4 in one chromosome and VviSVP5* and *VviSVP4 in another*. The 4 genes had similar expression, with the exception that *VviSVP5* and *VviSVP3* were more abundant in pollen and seed than in other reproductive organs.

Other genes belonging to the same subclasses had identical expression, and therefore could have redundant roles. This was the case for *VviSOC1a* and *VviSOC1b*; *VviAP1* and *VviAGL6b* as well as *VviBS2* and *VviBS1*. When genes showed co-expression only on the basis of available RNAseq data, the results were considered less reliable, since it was based on 11 conditions, versus 246 for a gene present in all microarrays. This was the case of *MADSD1a*, *MADS1G2d*, *MADS1G1c* and *MADS1G2f* on one side; *VviSVPS5* and *MADS1G1h*; *MADS1A3f* and *MADS1G2g*; *MADS1G2c, VviPI* and *MADS1A3c*, as well as *VviFUL1* and *MADS1A3i*. Finally, amongst genes that did not belong to the same clade and for which there was enough expression data collected, *VviSVP2* and *VviSOC1c* were of interest, beside sharing the same expression pattern, they were strictly expressed in vegetative tissues and not in reproductive tissues. Additionally, *VviSEP1*, *VviAG2*, *VviAG1* and *VviSEP3* shared the same expression patterns that are strictly limited to reproductive tissues. None of the MIKC* genes were co-expressed with any other MADS-box genes (Fig. 10).

## Discussion

We have performed an exhaustive analysis of MADS-box genes on the 12x grapevine genome based on the isolation of the complete set of genes identified in PN40024. In addition to public functional annotations, we flagged some regions that might represent functional MADS-box genes in other areas of the genome. This supposes the identification of 90 functional genes what adds 37 new genes to previous studies [38]. Chromosome localization, gene structure, phylogenetic analyses with other sequenced genome species and expression analysis allowed to propose an extended characterization of this gene family in grapevine and to draw hypothesis on the function of the yet undescribed genes.

### Grapevine MIKC^C^ –type II genes

A total of 42 MIKC^C^ –type II genes distributed in 13 subfamilies were found in grapevine, representing a similar number to what has been described in other plant species. These numbers include the addition of ten new genes to the previous descriptions of this group. Interestingly, we did not found putative one-to-one orthologs for these 10 new genes in other plant systems as detected for the remaining MIKC^C^ genes in previous analyses [37]. Grapevine shows a notable expansion of the SVP subfamily as has been described in other woody species [31, 45]. In addition, the new members in the VviSVP subfamily (*VviSVPS 1* to *5*) showed special features not yet found in any other species. The identification of new genes could result from our more thorough analysis over a 12x genome which, in comparison to automatic annotation methods, permits the isolation of genes with low similarity to other species and not strongly expressed, as automatic method rely on transcript data (EST, RNAseq) and sequences from other species.

The most significant data among the new genes found were the sequences called *VviSVPS* that appeared in the VviSVP subfamily. The MADS-box of VviSVPS proteins are highly similar to VviSVP and they were named in this way because of this similarity. They also showed similarities with the tomato *JOINTLESS* [46] that controls flower abscission. *VviSVPS* genes are located near *VviSVP* genes in grapevine. Three of these sequences (*VviSVPS1*, *VviSVPS2* and *VviSVPS3*) map linked to *VviSVP4* on chromosome 3 and showed a singular pattern of expression, partially overlapping with the expression of *VviSVP* genes but also expressed in other tissues. The same happened with sequences *VviSVPS4* and *VviSVPS5* which are linked to *VviSVP3* on chromosome 15. *VviSVPS* genes are however much shorter than *VviSVP* and *JOINTLESS*. The encoded proteins only contain the MADS-box and a short sequence of about 30 aminoacids that permits to discriminate VviSVPS2 and VviSVPS3 on one hand and VviSVPS1, VviSVPS4 and VviSVPS5 on the other hand (Fig. 4). Compared with grapevine *VviSVP* genes, they seem to lack a large part of the I region but showed homology at the end of the I region and the beginning of the K domain (Fig. 4). The conservation of the C-terminal small non-MADS-box sequence within the two groups indicates that this region is probably of functional interest. In addition, their specific expression pattern also suggests that these genes are probably functional. Short MADS-box genes have also been found in other species. Notably, one of them seems to be associated with dormancy in leafy spurge [47], whose expression could be in agreement with the expression pattern observed for *VviSVPS2*, *VviSVPS4* and *VviSVPS5* in grapevine. Short MADS-box proteins as the VviSVPS (<100 amino acids) and with sequence similarities to them were also detected in *Arabidopsis, Prunus persica, Ricinus communis, Citrus clementina, Populus trichocarpa, Glycine max, Citrus sinensis and Witheringia coccoloboides* in their respective predicted automatic annotations. *VviSVPS* genes had no homologs in the monocot species considered suggesting that duplication events giving rise to these new genes were rather recent and might actually play a role in dicot-specific developmental mechanisms. Their wide range and diversity of expression suggest that those mechanisms are rather diverse and establishment of their biological function will require further studies. The I and K domains are important for dimerization, The K-domain promotes dimerization via forming amphipathic helices, which interact with those of another K-domain-containing protein [5]. The I domain influences the specificity of DNA-binding dimer formation [1]. In VviSVPS, I-region is twice shorter than usual, and K-domain consists of 10–14 amino acids compared to 70–100 amino acids K-domain in MIKC proteins (Fig. 4), i.e. only 3–4 coils are formed instead of 20–30 coils in full-length K-domain alfa-helix. The proofs of their expression and their conservation in other species tends to indicate that they are functional, however, whether or not these proteins form complexes with other MADS-box proteins is unclear. A similar role could also be proposed for *VviTM8b*, although the truncated protein only contains the MADS-box and the expression of the corresponding gene could not be detected.

Regarding *VviAGL6b*, it showed a decreasing level of expression along inflorescence development but increased expression along tendril development [48] suggesting a possible involvement in development of this organ. The expression data (Additional file 2) indicate that the *VviAGL6a* probe set in the Grapegen array might have been compromised since expression was never detected in this platform, while it is clearly expressed in many tissues. Nevertheless its expression seems to be low in tendrils. Thus a new function acquired in this subfamily for tendril development was suggested for *VviAGL6b* [48], as was also proposed for other members of the AP1/FUL subfamily in grapevine [37] where additional subfamily members also show a differential pattern of tendril expression.

### Grapevine MIKC^*^–type II genes

The grapevine genome contained six genes in this group belonging to the two previously defined clades (S and P) [27, 28], that are typical of euphyllophytes (angiosperm, gymnosperms and ferns). Interestingly, clade 2, that included grapevine *MADSD2* genes, also included *AGL33*, an Arabidopsis gene difficult to classify and not assigned to either S or P clades but considered within the MIKC^*^subgroup, [26]. *MADSD2a* the closest grapevine gene to *AGL33* in the phylogenetic tree did not show homology to it or to genes from other species with the exception of poplar. This grapevine gene was clearly expressed in heterogeneous tissues indicating a possible ubiquitous role. The other grapevine gene in clade 2, *MADSD2b*, located alone in chromosome 17, corresponds to a single copy in every species except in strawberry and its Arabidopsis ortholog is *AGL65.* Its abundant and generalized expression is coincident with the expression behavior of *AGL65* according to TAIR (https://www.arabidopsis.org/servlets/TairObject?name=AT1G18750&type=locus).

In clade 1 there were three grapevine genes, *MADSD1a*, *MADSD1b* and *MADSD1c. MADSD1a* had a high number of one-to-one orthologs in other species and was expressed in flower and pollen, being a good candidate for having a functional role in pollen development. *MADSD1b* and *MADSD1c* are linked on the same chromosome and have similar patterns of ubiquitous expression suggesting that they could be functionally redundant. Arabidopsis genes within this clade (*AGL66* and *AGL104)* seem to function redundantly in pollen development since their loss has significant effects on pollen performance but only when both AGL66 and AGL104 MADS-box containing complexes are reduced [30]. Thus, we could think of a possible equivalent role for the complex (MADSD2b/MADSD2c*) /* MADSD1a as complexes AGL65/AGL104 or AGL65/AGL66 role in Arabidopsis pollen development. This is supported by their expression pattern since although *MADSD1a* and *MADSD2b/MADSD2c* have quite different patterns of expression they share the feature of peaking in pollen.

Arabidopsis and rice MIKC^*^ genes are almost exclusively expressed in pollen [49–51] with the exception of the *AGL67* gene which is expressed in embryos and is candidate for regulating aspects of late embryo development [29]. However, grapevine MIKC^*^genes are expressed outside male gametophytes with the exception of *MADSD1a* that was only detected in flower and pollen, which could suggest additional biological functions for these genes. Similarly, expression of P-clade genes was detected in sporophytic tissues in Prunus [31], in the female gametophyte in lycophytes as well as on hermaphrodite gametophytes in the fern Ceratopteris [27], suggesting that MIKC* genes could also function outside the male gametophyte in other systems. The basal angiosperm Eschscholzia [27] contains two MIKC^*^genes highly expressed in pollen with the one belonging to the P clade also expressed in sporophytic tissues. In Arabidopsis, heterodimers seems to exist only in pollen [30], suggesting a role of these genes also restricted to male gametophyte development in spite of the expression of the P clade gene. This could be also the case of grapevine with a broad expression of *MADSD2b* but with a more restricted expression for their S putative partners.

Finally, clade 3 contained a single grapevine gene, *MADSD3a*, which encodes truncated proteins with the MADS-box domain followed by five amino acids. *MADSD3a* is expressed in berry and seed. Coexpression analysis (Fig. 10) did not allow to identify clear potential heterodimers based on gene expression, although *MADSD3a* had similar expression pattern as *MADSD2a,* with high expression in seeds which could suggest a putative role in seed development, as has been found for *AGL67* in Arabidopsis. These data indicate that short MADS-box genes also exist in the MIKC* subgroup as has been observed for the MIKC^C^ genes.

### Grapevine type I MADS-box genes

We have identified 42 type I MADS-box genes in grapevine, 23 belonging to Mα-type I and 19 to Mγ-type I genes. Our analyses showed that no gene could be classified as Mβ in grapevine although a reduced clade of two genes might cover their biological roles. These genes numbers were very similar to those described in Arabidopsis with Mα (25 genes) and Mγ (16 genes) [26], although Arabidopsis displays a large group of Mβ genes (20). Type I genes number is variable among plant species without clear homologies within subclasses and this is also the case in grapevine [4, 33]. Our phylogenetic analysis showed the presence of grapevine-specific clades both in the Mα and Mγ-subclasses with only a few genes having clear homologs in other species. Gene clustering on chromosome location for many of these genes is also in agreement with their proposed origin through segmental duplications.

### The Mα-type I genes

Three clades were identified in grapevine MADS1A1 and MADS1A2, that are phylogenetically related and MADS1A3. In Arabidopsis, the Mα type I genes are separated into two groups according to their expression. The first group contains genes distinctly expressed while the genes in the second group are weakly expressed. In the first group, two clades with differential expression pattern were additionally found [32]. In grapevine, the first clade, MADS1A1, contained the genes showing the highest homology with this first clade of the first group of Arabidopsis (Fig. 7). This conservation was also observed in other plant systems. This clade in Arabidopsis contains genes with a proposed functional role in central cell development, endosperm development and early embryo sac and seed development (*DIA/AGL61* [52], *AGL62* [53], *AGL23* [54]). The expression detected for *MADS1A1* genes in grapevine is very low, probably due to expression of these genes in few and specific cells, never evaluated in grapevine and during short times along development. No Plant Ontology was attributed to any genes, but expression higher than the background noise were observed for three genes (*MADS1A1a*, *MADS1A1c* and *MADS1A1e*). The expression of *MADS1A1a* and *MADS1A1c* in berry and first stages of seed development (mainly in the case of *MADS1A1a*), and *MADS1A1e* in seeds would be in agreement with a role in seed development similarly to what has been proposed for their Arabidopsis homologs. Out of five of these genes that clustered on chromosome 5, only two might be expressed. This could be a consequence of segmental duplication that can derive in non-functionalization, as has also been observed in Arabidopsis and other plant systems. This was one of the few groups of grapevine type I genes that showed clear homology with genes from other plant species (Fig. 7), suggesting they might regulate conserved functions during reproductive development.

Regarding the second clade of grapevine genes, MADS1A2, their expression is generally very low except for two genes that are clearly expressed. Both *MADS1A2a* and *MADS1A2* were expressed in a diversity of tissues, senescing leaves, winter buds, post véraison berry tissues, seedling and pollen. In general, *MADS1A2* genes showed only little homology to genes from a few other species (papaya, soybean, medicago, tomato and poplar). The MADS1A2 clade do not seem to have any counterpart in Arabidopsis and cannot be related to the second group of Arabidopsis Mα-type I genes composed by genes weakly expressed in the female gametophyte. Interestingly, Arabidopsis genes belonging to the clade without grapevine counterparts were the ones interacting with Mβ genes, a group of genes also missing in grapevine. Considering that Mβ genes seem to be absent or partially absent in several species (Eucalyptus, soybean, medicago and grapevine), it is tempting to speculate the existence of alternative regulatory mechanisms for the development of the female gametophyte in plants, including grapevine. The third clade of Mα-type I genes in grapevine, MADS1A3, is phylogenetically related in the species analysis to the second clade of the first group of Mα-type I genes of Arabidopsis but do not show specific gene homologies. Grapevine genes also show reduced homology with other species, with the exception of *MADS1A3a* (and *MADS1A3e* to a lesser extend). In the species analysis, *MADS1A3* genes are in the same clade than the four Arabidopsis paralogs *AGL57*, *AGL58, AGL59* and *AGL64* which are expressed in the embryo and the peripheral endosperm [32]. We did not detected expression for *MADS1A3a, MADS1A3b, MADS1A3c* and M*ADS1A3d* but *MADS1A3e* was expressed in older tissues (senescing leaves, late ripening) and *MADS1A3f* and *MADS1A3g* only show signs of expression slightly over background noise.

### The Mγ-type I genes

Three clades were distinguished within this subclass, MADS1G1, MADS1G2 and MADS1G3. The first clade MADS1G1 is grapevine-specific (Fig. 8). All these grapevine MADS-box genes, except *MADS1G1d*, were identified for the first time in this work. Thus, there is no microarray data available to analyze their expression. RNAseq experiments detected no expression for *MADS1G1a, MADS1G1b, MADS1G1e* and only traces for *MADS1G1d* and *MADS1G1e*. By contrast, *MADS1G1f, MADS1G1g* and *MADS1G1h* were clearly expressed in shoot tips. More detailed studies will be required to address the possible biological function of this group of genes. Genes within the other group, *MADS1G1i* and *MADS1G1j*, were already identified in the grapevine genome. These two genes are linked in the genome and co-expressed with *MADS1G2a* appearing to be flower-specific. *MADS1G1j* is also the only gene displaying homology with genes in other species (apple, MDP0000753870).

Although no gene could be classified as Mβ-type I in grapevine, *AGL47* and *AGL82*, a subgroup of Arabidopsis Mβ-type I genes were located closely to the MADS1G1 clade in the phylogenetic tree (Fig. 8). *AGL47* was expressed during early megagametogenesis and *AGL82* in central cell [32]. Although these organs and cell types were not analyzed here, expression of *MADS1G1i* and *MADS1G1j* in flowers could suggest that they might fulfill the role of the Mβ-type I subclass in grapevine.

In the second clade (MADS1G2) there was a group of genes, *MADS1G2a* to *MADS1G2g*, clustering together and a single gene, *MADS1G2h*, with homology to the Arabidopsis genes belonging to the *AGL34, AGL35, AGL36, AGL37, AGL80, AGL86, AGL90* and *AGL92* group of expression, all involved in endosperm development. Mγ-type I genes are preferentially expressed in the developing seed in Arabidopsis, whereas *AGL80* is predominantly expressed in the central cell [55]. Multiple *MADS1G2* genes have *AGL80* as best match with *MADS1G2h* having the highest homology (homology percentage of 65 %, compared to 50 % for the others). *MADS1G2h* also had one-to-one orthologs in all dicot species analyzed, but none in monocots. AGL80 together with AGL61 (DIANA) are involved in central cell formation in the Arabidopsis embryo sac [56]. Interestingly, no grapevine homologs were identified for *AGL37 (PHERES1),* functionally characterized and involved together with *AGL62* in endosperm development. The group of grapevine *MADS1G2a* to *MADS1G2g* genes had an expression pattern restricted to seed in several cases although other genes were expressed in earliest reproductive stages (flower and inflorescences). In addition, the putative *AGL80* homolog *MADS1G2h*, was expressed in post-harvest berry and seed.

The third clade (MADS1G3) contained a single Mγ gene, *MADS1G3a*, which belonged to a clade with Arabidopsis genes *AGL95, AGL96* and *AGL48. MADS1G3a* did not share homology with any gene in the analyzed species and was slightly expressed in seeds. *MADS1G3a* might be the gene fulfilling the role of these Arabidopsis genes whose biological roles were described as redundant in embryo development [32].

In summary, grapevine type I genes include a few conserved members that could be crucial for embryo and endosperm development in parallel to the role of Arabidopsis *AGL80* the putative ortholog of *MADS1G2h* and *AGL61* and *AGL62*, putative orthologs of MADS1A1 clade genes. Processes related to central cell, embryo and endosperm development could be conserved under the same regulatory networks. Regarding the development of the female gametophyte, which in Arabidopsis is controlled by interaction between the second group of Arabidopsis Mα-type I genes and the Mβ–type I genes, grapevine genes are less conserved since no homologous to those Mα-genes were identified and Mβ genes might be absent or only represented by two members in grapevine.

## Conclusions

Our identification of MADS box proteins in the grapevine genome revealed, for this specific gene family at least, that automatic approaches were limited in gene prediction since most of type I genes had not been identified previously. Our genomic analysis of MADS-box genes in grapevine allowed the discovery of genes belonging to the BS, AGL17, AGL6 and TM8 subfamilies that had no homologs in other plant species. In addition, five sequences related to the VviSVP subfamily, named as VviSVPS could represent a new type of MADS-box genes not yet characterized in other plant systems.

Characterization of the MIKC*-subgroup confirm the proposed existence of S and P clades genes although the grapevine genome seems to have less redundancy in the P clade with only two members. Expression of these grapevine genes was detected in the male gametophyte but also in other tissues which support additional roles outside pollen development.

We have extensively described grapevine type I genes for the first time. We identified two subclades of Mα-type I and three subclades of Mγ-type I genes, but no genes could be clearly classified as Mβ in grapevine. Phylogenetic analysis among species showed that only few members of type I genes have clear homologs in other plant species and that grapevine-specific clades appeared both in the Mα and Mγ-subclasses. Comparing with Arabidopsis and other species type-I genes, we observed conservation of genes that could be crucial for development of central cell, embryo and endosperm. Further functional analysis will be required to understand the biological role of this complex gene family in grapevine.

## Methods

### Identification of MADS-box genes

Genes previously identified as MADS-box genes [40] were blasted (blastp and tblastn) against the grapevine genome 12x.2 (https://urgi.versailles.inra.fr/Species/Vitis/Data-Sequences/Genome-sequences), the non redundant list of genes in [40] and the COST annotation gene set available at the ORCAE website (http://bioinformatics.psb.ugent.be/orcae/). Results from different analyses were manually cross-check to identify the potential locus in the 12x.2 genome of known genes and potential new locus. The UGene [57] software was used to localize the gene model on the grapevine genome and test the structure.

### Structure analysis

The coding DNA sequences (CDS) were blasted (blastx) against the NCBI public database to compare the structures with other known MADS-box genes in other species and with the NCBI predictions for the grapevine genes. When discrepancies were observed, gene models were corrected using the Ugene software. Loci giving rise to genes that were not functional were eliminated from the list (list and position in Additional file 1). A GFF (General File Format) file with the MADS-box genes was designed, uploaded into the IGV software and the RNAseq data available (shoot tips, leave, flower inflorescence and seed tissues) in the laboratory were used to double-check the exon structure of the genes. Final models were uploaded in the *Vitis vinifera* ORCA database [36, 39]. Protein domains were directly retrieved from the post-upload analysis automatically performed in the ORCAE database on InterPro, PANTHER, COILS and Phobius [58]. COILS is a software program that compares a sequence to a database of known parallel two-stranded coiled-coils and derives a similarity score [59]. The protein domain modeling were performed with DomainDraw [60].

### Sequence alignment and phylogenetic analysis

Previously described Arabidopsis MADS-box genes [26] were retrieved from the TAIR database [61]. Phylogenetic analyses were conducted using MEGA6 [62]. Multiple sequence alignment was inferred using MUSCLE [63]. The evolutionary history was inferred by using the Maximum Likelihood method based on the JTT matrix-based model [64]. The bootstrap consensus tree inferred from 100 replicates was taken to represent the evolutionary history of the taxa analyzed [65]. Branches corresponding to partitions reproduced in less than 30 % of bootstrap replicates were collapsed. Initial trees for the heuristic search were obtained automatically by applying Neighbor-Join and BioNJ algorithms to a matrix of pairwise distances estimated using a JTT model, and then selecting the topology with superior log likelihood value. The coding data was translated assuming a Standard genetic code table. All positions with less than 95 % site coverage were eliminated. Genes were named according to [39] based on the distance homology with Arabidopsis genes.

### Comparison to other species

For sequence comparison with the MADS box genes from 16 plant species (Arabidopsis thaliana, *Brassica rapa, Carica papaya, Eucalyptus grandis, Citrus sinensis, Malus domestica, Prunus persica, Fragaria vesca, Glycine max, Medicago truncatula, Cucumis melo, Populus trichocarpa, Solanum lycopersicum, Zea mays, Sorghum bicolor, Oryza sativa*) they retrieved at http://planttfdb.cbi.pku.edu.cn in the “M-type (type 1 and delta)” and “MIKC^C^” sections. Orthologous genes in genomes from the 16 species were identified following the approached used by [35]. Each pair of predicted gene sets was aligned with the BLASTx algorithm, and only alignments with an *e*-value lower than 1e^−20^ and sequence homology higher than 40 % were retained. If a comparison was above that value the two genes were considered homologs. The percentage cutoff allowed lowering the weight of homology only on the MADS-box. Two genes, A from *Vitis* genome GV and B from genome GX, were considered orthologs if B was the best match for gene A in GX and A was the best match for B in GV, else genes were considered homologs. A phylogenetic tree was constructed with the “M-type” from these species with the same parameters as before.

### Expression analysis

Expression data were retrieved from 3 different microarray platforms (Affymetrix Genchip (16 k probesets) GrapeGen (21 k probesets),Vitis Nimblegen array (29 k probesets) and from our in-house RNAseq projects. Data normalization was performed on all the array of each platform (RMA normalization). After retrieving the values for the probesets corresponding to each gene, the values for the 3 or 4 replicates of the same condition were averaged to obtain a total of 256 conditions (organ, cultivar, treatment, platform). Based on expression data, a plant ontology ID was attributed to each gene if expression intensity in a tissue was above a defined threshold of absolute intensity for each platform (orange and red values in Fig. 9). Normalized signal intensity higher than 8 (log_2_ value) for microarray data, at least 10 reads for RNAseq data). For the coexpression analysis, in order to minimize the weight of the conditions that were performed numerous times without bringing pertinent information a preliminary cluster analysis was performed. Non redundant array were obtained by averaging the values of the probeset for the same gene of arrays that present a distance threshold of the MADS-box expression lower than 0.05 for hierarchical clustering (Pearson correlation, average linking). To compare the relative expression of the genes, a second cluster analysis was performed on the non redundant array with the same parameters. Genes considered as having the same profile should present a distance threshold between each other lower than 0.15. For cluster analysis, the value with a low intensity in the microarrays data (green values in Fig. 9) where variability is mainly caused by noise were smoothed to a basal log_2_ value of 5.

## Additional files

Additional file 1:
**Chromosome position of the MADS-box genes that do not seem to be functional.** Bold: The lack of detection of a functional gene might be due to incomplete data in the assembly or the gene might be functional in a different cultivar or *Vitis* species. (DOCX 13 kb) Additional file 2:
**Complete annotation of grapevine MADS-box genes.** These data were also been deposited at the Grapevine annotation platform. http://bioinformatics.psb.ugent.be/orcae/ (XLSX 34 kb) Additional file 3:
**Molecular phylogenetic analysis by Maximum Likelihood method between Grapevine and Arabidopsis MADS-box deduced proteins.** The evolutionary history was inferred using the Maximum Likelihood method based on the JTT matrix-based model [64]. The tree with the highest log likelihood (−18560.7870) is shown. The percentage of trees in which the associated taxa clustered together is shown next to the branches. Initial tree(s) for the heuristic search were obtained by applying the Neighbor-Joining method to a matrix of pairwise distances estimated using a JTT model. The analysis involved 187 amino acid sequences. All positions with less than 95 % site coverage were eliminated. That is, fewer than 5 % alignment , missing data and ambiguous bases were allowed at any position. There were a total of 92 positions in the final dataset. Evolutionary analyses were conducted in MEGA6 [62]. (PDF 859 kb) Additional file 4:
**Phylogenetic tree of MIKC* and type I MADS-box proteins in plants, branches were not collapsed.** Green: grapevine deduced proteins. Pink: Arabidopsis proteins. (PDF 357 kb) Additional file 5:
**References of the microarray experiments used in the expression analysis.** (DOCX 37 kb) Additional file 6:
**Original expression data (RMA normalized) from the expression experiments described in Additional file** 4. (XLSX 140 kb)
