# Supplementary material for: Structural and functional annotation of the MADS-box transcription factor family in grapevine
Source: BMC Genomics. 2016 Jan 27;17:80. doi: 10.1186/s12864-016-2398-7 (PMC4729134; doi:10.1186/s12864-016-2398-7)
Supplement: Additional file 1: — Chromosome position of the MADS-box genes that do not seem to be functional. Bold: The lack of detection of a functional gene might be due to incomplete data in the assembly or the gene might be functional in a different cultivar or Vitis species. (DOCX 13 kb) [file 12864_2016_2398_MOESM1_ESM.docx]

**Additional file 1**. Chromosomal position of MADS-box genes that did not appear to belong to a functional gene. Bold: Lack of detected functional structure could be due to incomplete sequence data in the assembly or the gene might be functional in another cultivar.

| Chromosome | Position |
| --- | --- |
| chr00 | **complement(24505387..24505650)** |
| chr01 | **complement(join(1600202..1600581,1600693..1600739,1600861..1600902,1601109..1601272,1601382..1601450,1602332..1602517))** |
| chr01 | **complement(19055389..19055921)** |
| chr03 | **10790483..10790644** |
| chr05 | **complement(21835788..21836023)** |
| chr08 | **20140977..20141178** |
| chr08 | **complement(join(4946606..4946904,4951644..4951681,4951687..4951937,4951940..4952329))** |
| chr12 | **12634867..12635396** |
| chr15 | **complement(17739948..17740139)** |
| chr15 | **17821308..17821499** |
| chr15 | **complement(17864506..17864690)** |
| chr15 | **complement(17931826..17932017)** |
| chr15 | **complement(17967523..17967714)** |
| chr19 | **join(13123834..13124068,13124070..13124198,13124201..13124382,13124682..13124735)** |
| chr19 | **complement(join(15142596..15142904,15142909..15143133))** |
| chr01 | 12645816..12646159 |
| chr02 | 13267758..13268257 |
| chr02 | 13272418..13272924 |
| chr02 | complement(14549891..14550040) |
| chr02 | 13287079..13287523 |
| chr01 | complement(join(1341372..1341569,1341682..1341759)) |
| chr01 | join(1342308..1342376,1342486..1342689) |
| chr01 | complement(1603595..1603849) |
| chr01 | complement(1605904..1606143) |
| chr01 | complement(19018974..19019421) |
| chr03 | 6875891..6876037 |
| chr03 | 8685629..8685922 |
| chr03 | 8717078..8717707 |
| chr07 | complement(join(20813741..20813825,20813907..20814058,20814159..20814278,20814373..20814492,20814971..20815090,20815199..20815304,20816222..20816317)) |
| chr07 | complement(23556037..23556195) |
| chr07 | complement(25718247..25718485) |
| chr10 | complement(8683261..8683410) |
| chr12 | complement(19359186..19359387) |
| chr13 | 13806131..13806298 |
| chr14 | complement(4216493..4216713) |
| chr15 | 17982499..17982633 |
| chr16 | 13328671..13329006 |
