# Supplementary figures and images for: Structural and functional annotation of the MADS-box transcription factor family in grapevine
Source: BMC Genomics. 2016 Jan 27;17:80. doi: 10.1186/s12864-016-2398-7 (PMC4729134; doi:10.1186/s12864-016-2398-7)

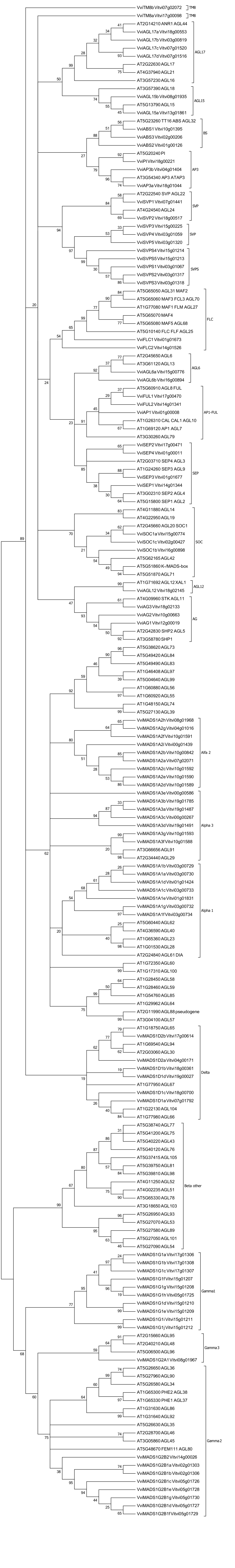

Supplement: Additional file 3: — Molecular phylogenetic analysis by Maximum Likelihood method between Grapevine and Arabidopsis MADS-box deduced proteins. The evolutionary history was inferred using the Maximum Likelihood method based on the JTT matrix-based model [64]. The tree with the highest log likelihood (−18560.7870) is shown. The percentage of trees in which the associated taxa clustered together is shown next to the branches. Initial tree(s) for the heuristic search were obtained by applying the Neighbor-Joining method to a matrix of pairwise distances estimated using a JTT model. The analysis involved 187 amino acid sequences. All positions with less than 95 % site coverage were eliminated. That is, fewer than 5 % alignment , missing data and ambiguous bases were allowed at any position. There were a total of 92 positions in the final dataset. Evolutionary analyses were conducted in MEGA6 [62]. (PDF 859 kb) [file 12864_2016_2398_MOESM3_ESM.pdf]
