# Supplementary material for: Structural and functional annotation of the MADS-box transcription factor family in grapevine
Source: BMC Genomics. 2016 Jan 27;17:80. doi: 10.1186/s12864-016-2398-7 (PMC4729134; doi:10.1186/s12864-016-2398-7)
Supplement: Additional file 5: — References of the microarray experiments used in the expression analysis. (DOCX 37 kb) [file 12864_2016_2398_MOESM5_ESM.docx]

**Additional File 4**. Microarray experiments used in the expression analysis.

| **Plexdb ID** | **GEO/ Arrayexpress** | **Tissue** | **Cultivar** | **Publication** |
| --- | --- | --- | --- | --- |
| **VV1** | GSE31594 | Shoot tip | Cabernet Sauvignon | (Tattersall et al., 2007) |
| **VV2** | GSE31677 | Shoot tip | Cabernet Sauvignon | (Cramer et al., 2007) |
| **VV3** |  | Berry skin pulp and seed | Cabernet Sauvignon | (Grimplet et al., 2007) |
| **Vv5** |  | Berry | Cabernet Sauvignon, Chardonnay | (Deluc et al., 2007) |
| **Vv7** |  | leaf | Camenere | (Espinoza et al., 2007) |
| **Vv9** | GSE31675 | berry | Cabernet Sauvignon |  |
| **Vv10** | GSE17502 | bud | Seyval, V. Riparia | (Sreekantan et al., 2010) |
| **Vv11** | GSE31674 | berry | Pinot Noir | (Pilati et al., 2007) |
| **Vv12/ Vv13** | GSE6404 | Leaf | Cabernet Sauvignon V. aestivalis | (Fung et al., 2008) |
| **Vv14** | GSE12842 | Inflorescence | Chardonnay, Mazoni | (Albertazzi et al., 2009) |
| **Vv15** | GSE11406 | berry | Cabernet Sauvignon | (Lund et al., 2008) |
| **Vv16** | GSE31664 | Berry skin | Cabernet Sauvignon | unpublished |
| **Vv17** | GSE31662 | Berry skin | Cabernet Sauvignon | unpublished |
| **Vv19** | GSE11857 | Leaf | V. rotundifolia | unpublished |
| **Vv28** | GSE31660 | Leaf | Cabernet Sauvignon | (Vega et al., 2011) |
| **Vv29** | GSE27180 | Leaf | Touriga Nacional | (Carvalho et al., 2011) |
| **Vv31** | GSE29948 | Aerial tissues | Freedom | (Tillett et al., 2012) |
| **Vv32** |  |  | Tempranillo | unpublished |
| **Vv33** | GSE41206 | Berry, pulp, skin | Muscat Hamburg | (Lijavetzky et al., 2012) |
| **Vv36** | GSE42345 | Bud, flower ,inflorescence | Tempranillo | (Díaz-Riquelme et al., 2012) |
| **Vv45** | GSE28779 | Berry | Trincadeiro | (Fortes et al., 2011) |
| **Vv46** | GSE43043 | Berry | Muscat Hamburg | (Carbonell-Bejerano et al., 2013) |
|  | E-MEXP-2541 | Leaf | Malbec | (Pontin et al., 2010) |
|  | GSE36128 | Tissue atlas |  | (Fasoli et al., 2012) |
