# Supplementary material for: Structural and functional annotation of the MADS-box transcription factor family in grapevine
Source: BMC Genomics. 2016 Jan 27;17:80. doi: 10.1186/s12864-016-2398-7 (PMC4729134; doi:10.1186/s12864-016-2398-7)
Supplement: Additional file 4: — Phylogenetic tree of MIKC* and type I MADS-box proteins in plants, branches were not collapsed. Green: grapevine deduced proteins. Pink: Arabidopsis proteins. (PDF 357 kb) [file 12864_2016_2398_MOESM4_ESM.pdf]

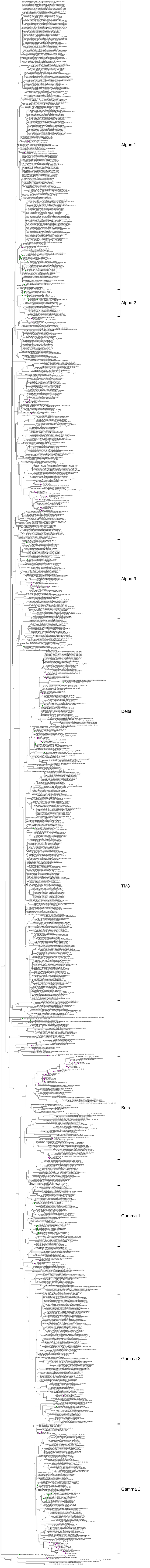
Alpha 1
Alpha 2
Alpha 3
Delta
TM8
Beta
Gamma 1
Gamma 3
Gamma 2
